# Excreted secreted products from the parasitic nematode *Steinernema carpocapsae* manipulate the *Drosophila melanogaster* immune response

**DOI:** 10.1038/s41598-022-18722-7

**Published:** 2022-08-20

**Authors:** Kirah Jones, Ghada Tafesh-Edwards, Eric Kenney, Duarte Toubarro, Nelson Simões, Ioannis Eleftherianos

**Affiliations:** 1 Department of Biological Sciences, The George Washington University, Washington, DC 20052 USA; 2 CBA and Faculty of Sciences and Technology, University of Azores, Rua Mãe de Deus no13, 9500-321 Ponta Delgada, Portugal

**Keywords:** Antimicrobial responses, Immune evasion, Infection, Inflammation, Innate immunity

## Abstract

*Steinernema carpocapsae* is an entomopathogenic nematode (EPN) that rapidly infects and kills a wide range of insect hosts and has been linked to host immunosuppression during the initial stages of infection. The lethal nature of *S. carpocapsae* infections has previously been credited to its symbiotic bacteria; however, it has become evident that the nematodes are able to effectively kill their hosts independently through their excretion/secretion products (ESPs). Here we examined how the adult *Drosophila melanogaster* immune system is modulated in response to *S. carpocapsae* ESPs in an attempt to ascertain individual pathogenic contributions of the isolated compound. We found that the *S. carpocapsae* ESPs decrease the survival of *D. melanogaster* adult flies, they induce the expression of certain antimicrobial peptide-encoding genes, and they cause significant reduction in phenoloxidase enzyme activity and delay in the melanization response in males flies. We also report that *S. carpocapsae* ESPs affect hemocyte numbers in both male and female individuals. Our results indicate the manipulative role of EPN ESPs and reveal sex-specific differences in the host response against nematode infection factors. These findings are beneficial as they promote our understanding of the molecular basis of nematode pathogenicity and the parasite components that influence nematode-host interactions.

## Introduction

The *Drosophila* immune response is composed of conserved signaling pathways and mechanisms that expand to vertebrate animals, making *Drosophila* a suitable model for studying the innate immune system in relation to human disease ^1,2^. Flies and humans share similar proteins and processes for pathogen recognition, signal induction and transduction, microbial clearance, and regulation of transcription factors for the expression of immune effectors. These properties enable *Drosophila* to act as a biological interface to understand how the conserved host innate immune reactions dictate the progression of disease, which will help us design novel treatments to combat infectious pathogens ^3,4^.

The *Drosophila* innate immune system discriminates between self and non-self through specialized pattern recognition receptors (PRRs) that survey the internal environment for distinct molecular patterns that are conserved across pathogenic groups ^5,6^. The epithelial immunity at barrier sites functions similarly between flies and humans to protect against invasion including the shared presence of a diverse microbial flora ^7,8^. When physical barriers are breeched, *Drosophila* employs both cellular and humoral responses to mitigate infection ^9,10^. Hemocytes, predominately plasmatocytes, immediately implement phagocytosis through direct binding of their PRRs to pathogen-associated molecular patterns (PAMPs) or indirectly with cell aggregation, marking the foreign particle for opsonization ^11^. Plasmatocytes closely resemble tissue-resident macrophages found in vertebrates because of their embryonic origin, ability to differentiate, and phagocytic function that parallels the two myeloid lineages observed in vertebrates ^12^. Once PAMPs are detected, hemocytes play a critical role in relaying signals of infection to surrounding tissues through paracrine secretion, which induces humoral responses through the Nuclear factor-kappaβ (NF-κβ) signaling pathways Toll and Immune deficiency (Imd), or the Janus kinase/signal transducer and activator of transcription (JAK-Stat) pathway. This in turn promotes the production of fat body derived antimicrobial peptides (AMPs) as well as other stress response proteins that dominate the hemolymph several hours after infection ^13–16^. In addition, the transforming growth factor beta (TGF-β) pathway is primarily involved in the regulation of larval development in *Drosophila* and the control of conserved immune functions and mechanisms with anti-inflammatory and tissue repair properties ^17^. Interestingly, our recent work has uncovered the participation of TGF-β signaling in the *Drosophila* immune response against parasitic nematode infection and wounding ^18–20^. The third component of the *Drosophila* host defense is the prophenoloxidase (PPO) cascade, which connects cellular and humoral immunity and is responsible for creating a cytotoxic environment to combat microbial growth and sealing off wounds to inhibit further invasion ^21,22^.

Entomopathogenic nematodes (EPNs) are able to infect and rapidly kill a wide range of insect hosts within the first few hours of infection. EPNs are classified as parasitic due to their infective juvenile (IJ) stage in which they are developmentally arrested and require a host to resume their life cycle ^23,24^. *Steinernema carpocapsae* parasitic nematodes are specifically adapted for highly mobile insects and they reside stationary in the soil where they stand upright waiting to ambush and strike potential hosts crossing their path ^25^. Upon invasion through natural openings, *S. carpocapsae* nematodes release their mutualistic *Xenorhabdus nematophila* bacteria, which produce virulence factors that contribute to the host’s demise ^26^. After multiple rounds of nematode replication and feeding on the host tissues, the nematode offspring emerge from the insect cadaver in search for their next host. Two main strategies employed by EPNs to evade the host immune system include molecular mimicry and modulation of the host immune response ^27^. In the early stages of infection, the nematodes primarily rely on mimicry by displaying surface molecules that interfere with hemocyte surveillance to prevent recognition. For instance, *S. carpocapsae* IJs have been shown to express a protein in their epicuticle that mimics host receptors ^28^. Direct manipulation of the host defense, on the other hand, involves the excretion/secretion of effector molecules. In this context, *S. carpocapsae* excretion/secretion products (ESPs) have been linked to host immunosuppression during the initial stages of invasion to promote a suitable growing environment for their mutualistic bacteria ^29,30^.

EPN pathogenicity is typically attributed to toxins and virulence factors that are released in conjunction with the *X. nematophila* mutualistic bacteria*,* but further investigation has shown that EPNs play an active role in the pathogenicity of the nematode-bacteria complex ^31^. Studies with axenic nematodes have demonstrated that ESPs retain their toxic effects causing insect death within the first two hours after injection, which suggests that the success of EPNs is independent of the action of their mutualistic bacterial partners ^32^. The composition of these ESPs includes proteases and protease inhibitors involved in tissue degradation as well as other small molecules and toxins that promote immune suppression ^31^. In particular, a serpin-like inhibitor isolated from *S. carpocapsae* ESPs functions to impair the integrity of clot fibers by suppressing phenoloxidase (PO) enzyme activity and consequently preventing the deposition of melanin to fortify hard clots, which are necessary for the nodulation and sequestration of invading microbes ^33^.

EPNs contain conserved orthologs which are maintained across vertebrate and human parasitic nematodes, rendering them ideal model organisms for conducting research in a safe and cost-effective manner to understand helminth-related diseases that negatively impact human health ^30,32,34^. The aim of this research was to examine how the adult *Drosophila* immune system is modulated in response to *S. carpocapsae* ESPs and determine the pathogenic contributions of the isolated molecules. This information will help us gain a better understanding of how nematode parasites impact the host immune system and potentially lead to the development of new approaches for combating infectious disease in humans and agricultural enemies by designing innovative pest management strategies to control pernicious insects.

## Results

### *Steinernema carpocapsae* excreted/secreted products are pathogenic to *Drosophila melanogaster* adult flies

**Figure 1:**
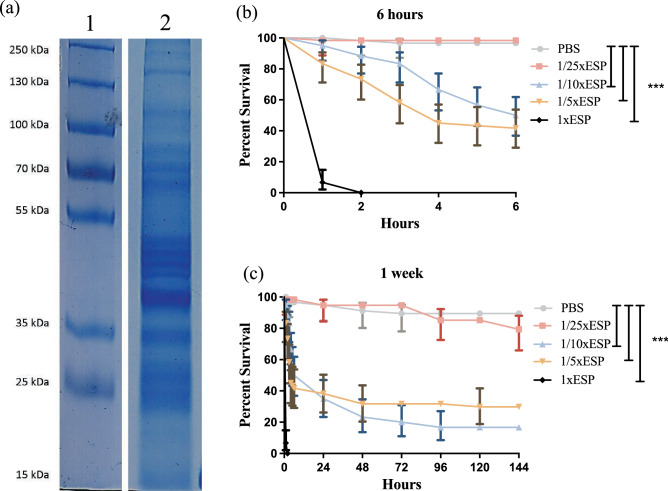
SDS-PAGE analysis of the *Steinernema carpocapsae* excreted/secreted products (ESPs) and survival of *Drosophila melanogaster* adult flies following injection with nematode ESPs. (**a**) Protein molecular weight marker (Plus Prestained Protein Ladder, 15 to 250 kDa, Thermo Scientific) (Lane 1) and ESPs of *S. carpocapsae* induced for 18 h in insect tissue homogenate (Lane 2). Cropped image; the original gel is presented in Supplementary Fig. 1. (**b**) Survival percentage of adult wild type Oregon-R flies within the first 6 h of injection with different concentrations of *S. carpocapsae* ESPs; 1xESP (4.25 μg/μl), 1/5xESP (0.85 μg/μl), 1/10xESP (0.43 μg/μl), or 1/25xESP (0.17 μg/μl) dilutions. Individuals injected with PBS were used as a control group. (**c**) Survival percentage of adult wild type Oregon-R flies over the course of 1 week (144 h) following injection with different concentrations of *S. carpocapsae* ESPs. Significant differences between survival curves were calculated using Log-rank (Mantel-Cox) Test, (***P < 0.0001).

Most proteins in ESPs of *S. carpocapsae* nematodes which were induced for 18 h with insect tissue homogenate ranged from 25 to 55 kDa in size (Fig. 1a and Supplementary Fig. 1). Initial trials were conducted to determine the effect of *S. carpocapsae* nematode ESPs on *D. melanogaster* fly survival. A series of dilutions was prepared and injected into *D. melanogaster* adults and survival percentages were estimated over the course of one week. We found that *S. carpocapsae* ESPs reduced the *D. melanogaster* survival rates with the greatest effects observed within the first six hours after injection, during which 93.3% of individuals treated with the full 1xESP concentration died within the first hour (Fig. 1b). Fly groups treated with the 1/5xESP and 1/10xESP concentrations experienced an average mortality of 58.3% and 50%, respectively, at 6 h post injection. After 24 h, the survival of both groups remained constant over the course of a week (Fig. 1c). Individuals treated with the 1/25xESP concentration were the only group that did not show a significant change in survival compared to the phosphate buffered saline (PBS) controls and was selected as the concentration for following experiments. These findings demonstrated the potent effects of *S. carpocapsae* ESPs on the survival ability of adult *D. melanogaster*.

### *Steinernema carpocapsae* excreted/secreted products induce the expression of antimicrobial peptide-encoding genes in *Drosophila melanogaster* adult flies

**Figure 2:**
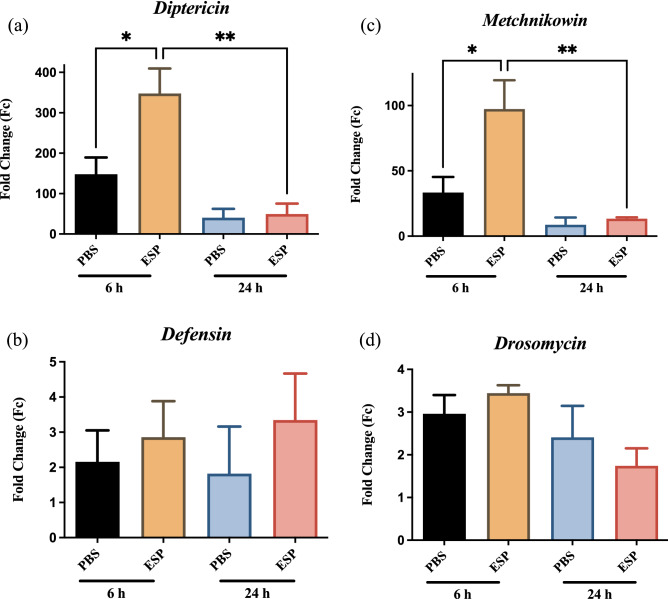
Antimicrobial peptide gene expression in *Drosophila melanogaster* injected with *Steinernema carpocapsae* nematode excreted/secreted products (ESPs). Oregon-R adult flies were injected with 3 ng of ESPs from *S. carpocapsae* nematodes or PBS as a control. Samples were collected at 6 and 24 h and following quantitative RT-PCR, mRNA values were normalized to a baseline 0-h time point and the housekeeping gene *RpL32.* Expression of (**a**) *Diptericin*, (**b**) *Defensin*, (**c**) *Metchnikowin*, and (**d**) *Drosomycin* (*P < 0.01, **P < 0.001; One-way ANOVA).

The transcriptional expression of AMPs associated with the Imd and Toll pathways were examined by RT-qPCR. We specifically assessed differences in expression levels at 6 and 24 h post injection, which correspond to the peak activity times for Imd and Toll signaling, respectively. Individuals injected with *S. carpocapsae* ESPs demonstrated a significant increase in *Diptericin* expression, a readout of the Imd pathway, at 6 h compared to PBS controls (Fig. 2a). Compared to the 6-h time point, the activity of the ESP group at 24 h showed a significant reduction in expression. Interestingly, we found no significant changes in the expression of *Defensin*, which is also regulated by the Imd pathway, among the experimental treatments at any of the tested time points (Fig. 2b). Next, we examined the expression of *Metchnikowin* and *Drosomycin*, which are regulated by the *Toll* pathway, and found that *Metchnikowin* (Fig. 2c), but not *Drosomycin* (Fig. 2d), displayed a significant increase in expression at 6 h after injection of nematode ESPs, which like *Diptericin* was significantly reduced after 24 h. These findings support the notion that the *S. carpocapsae* ESPs are able to induce both the Imd and Toll signaling pathways during early stages of the immune response.

### *Drosophila melanogaster* JAK/Stat and TGF-β signaling activities are unaffected by *Steinernema carpocapsae* excreted/secreted products

**Figure 3:**
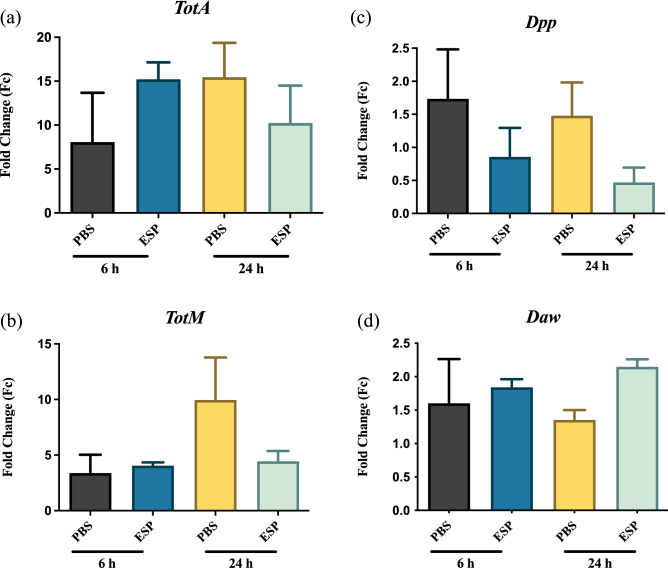
Expression of immune-related genes in *Drosophila melanogaster* injected with *Steinernema carpocapsae* nematode excreted/secreted products (ESPs). Oregon-R adult flies were injected with 3 ng of ESPs from *S. carpocapsae* nematodes or PBS as a control. Samples were collected at 6 and 24 h and following quantitative RT-PCR, mRNA values were normalized to a baseline 0-h time point and the housekeeping gene *RpL32.* Expression of the JAK-Stat regulated genes (**a**) *TotA*, (**b**) *TotM*, and the TGF-β regulated genes (**c**) *Dpp*, and (**d**) *Daw*.

We tested whether *S. carpocapsae* ESPs affect the signaling activity of other pathways that participate in the *D. melanogaster* immune response. Using RT-qPCR analysis, we found no significant changes in the expression of the JAK/Stat-regulated, stress response genes *TotA* and *TotM* between flies injected with the *S. carpocapsae* ESPs and those injected with PBS (Fig. 3a, b). Also, we observed that the *Dpp* ligand of the bone morphogenic protein (BMP) signaling branch of the TGF-β pathway ^17^, showed a trend of reduced expression for the group treated with the nematode ESPs compared to the control group, but the reduction was not statistically significant (Fig. 3c). Finally, there was no differential expression for *Daw*, which encodes a ligand in the Activin branch of the *D. melanogaster* TGF-β pathway ^17^ (Fig. 3d). Collectively, none of the JAK/Stat and TGF-β pathway related genes examined displayed differential expression in response to *S. carpocapsae* ESPs.

### *Steinernema carpocapsae* excreted/secreted products reduce the activity of phenoloxidase (PO) in *Drosophila melanogaster* adult flies

**Figure 4:**
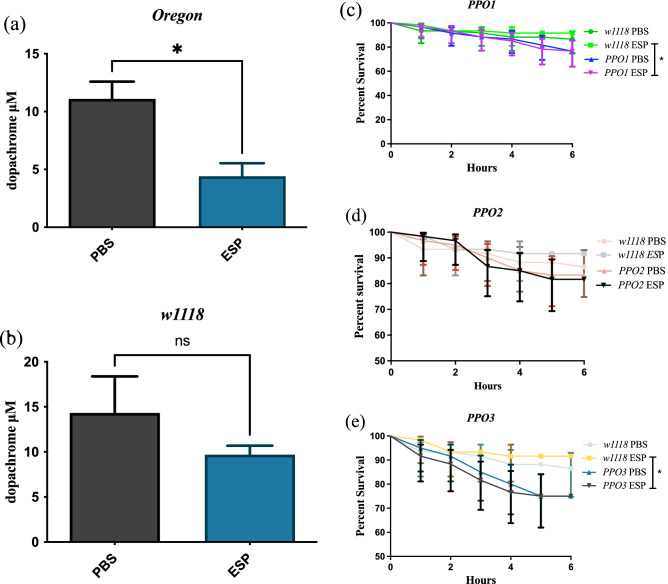
Effect of *Steinernema carpocapsae* excreted/secreted products (ESPs) on the *Drosophila melanogaster* phenoloxidase response. Adult flies of the (**a**) Oregon-R and (**b**) *w1118* lines were injected during separate trials with 3 ng of ESPs from *S. carpocapsae* nematodes or PBS as a negative control. One hour post injection, phenoloxidase activity was assessed through the reduction of L-DOPA into dopachrome (*P < 0.01; One-way ANOVA). Mutant flies for (**c**) *PPO1*, (**d**) *PPO2* and (**e**) *PPO3* genes were injected with nematode ESPs and individuals that received PBS served as a control group for each fly mutant line. The survival percentage of the PPO mutant lines was compared at 6 h after injection against the background line *w1118*. Significant differences between survival curves were calculated using Log-rank (Mantel-Cox) Test (*P < 0.01; ns: non-significant differences.

The effect of *S. carpocapsae* ESPs on PO activity was assessed in *D. melanogaster* Oregon-R and *w1118* lines using a previously established biochemical assay ^35^. We found that Oregon-R adult flies injected with the nematode ESPs had significantly reduced PO activity compared to PBS injected individuals (Fig. 4a). However, *w1118* adult flies injected with the nematode molecules had a non-significant downward trend of reduced PO activity compared to PBS treated controls (Fig. 4b). Using mutants for each of the three *PPO* genes in *D. melanogaster*, we performed survival experiments to test the survival rates of PPO deficient flies following injection with *S. carpocapsae* ESPs. *PPO1* and *PPO3* mutants treated with nematode ESPs showed a significant decrease in survival compared to their *w1118* background controls (Fig. 4c, e). However, this survival effect was not observed in *PPO2* mutant flies (Fig. 4d). These findings indicate that *S. carpocapsae* ESPs are capable of reducing the activity of the PO response in *D. melanogaster*, and functional *PPO1* and *PPO3* genes are important for the survival of adult flies against EPN infection factors.

### *Steinernema carpocapsae* excreted/secreted products decrease the melanization response in male *Drosophila melanogaster* adult flies

**Figure 5:**
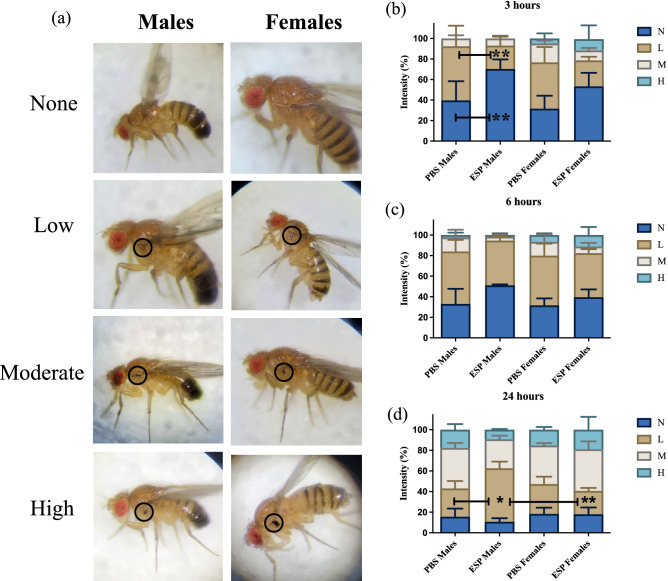
*Steinernema carpocapsae* nematode excreted/secreted products (ESPs) affect the melanization response in *Drosophila melanogaster*. (**a**) Qualitative categorization of the degree of melanization displayed in male and female adult flies; None = no visible signs of a melanin spot at the injection site, Low = visible melanin spot that is less than or equal to the size of the injection site and the pigment is translucent, Moderate = melanin spot that is equal to the size of the injection site with pigment semi-opaque to fully opaque, High = melanin spot that is greater than the size of the injection site with hyperpigmentation that is opaque. Adult flies of the Oregon-R line were divided into male and female groups and then injected with 3 ng of *S. carpocapsae* ESPs or PBS as a control and observed at (**b**) 3 h, (**c**) 6 h, and (**d**) 24 h for changes in melanization intensity (N = None, L = Low, M = Moderate, and H = High). *P < 0.05, **P < 0.01; Two-way ANOVA.

Next, we assessed the melanization response of *D. melanogaster* against the *S. carpocapsae* ESPs by observing the degree of melanin formation around the injection site in male and female flies over the course of a 24-h period. Melanization levels were classified as None, Light, Moderate, or High based on the size and intensity of black pigment melanin at the injection area (Fig. 5a). Within the first 3 h after injection, males treated with nematode ESPs had a significant delay in inducing the melanization response with the PBS control group containing 39.67% and 52.67% of individuals with no or light melanization compared to 70.33% and 22.67% for males injected with the nematode products (Fig. 5b). Females had no difference in melanization between the ESP and control groups at 3 h. Although not significant, it was interesting that none of the PBS-injected males and ESP-injected males contained any individuals with a high degree of melanization within the first 3 h compared to the female flies with frequencies of 5% and 11% for the PBS and ESP groups, respectively. At 6 h post injection, neither males nor females displayed significant differences in melanization between the experimental and control groups (Fig. 5c). At the 24-h time point, male flies maintained a significant difference in melanization with a higher percentage of ESP-injected individuals (52.0%) having light melanization compared to the control group (27.3%) (Fig. 5d). Likewise, there was a significant difference in the number of individuals with light melanization between males (52.0%) and females (22.67%) whereas the PBS injected males had similar frequencies to PBS injected females. By this point, male and female groups presented similar levels of high melanin pigmentation. These results suggest that *D. melanogaster* males have a delayed melanization response compared to female flies when challenged with *S. carpocapsae* ESPs. Both PBS and ESP treated males experience most of significant changes between 3–24 and 6–24 h whereas ESP treated females are more affected over 24 h compared to controls.

### *Steinernema carpocapsae* excreted/secreted products alter hemocyte numbers in *Drosophila melanogaster* adult flies

**Figure 6:**
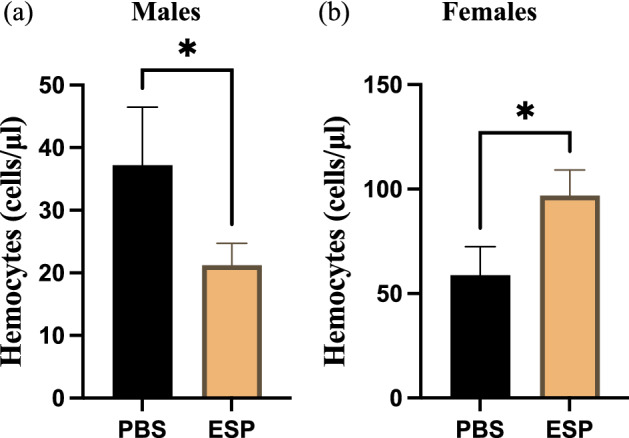
*Steinernema-carpocapsae* nematode excreted/secreted products (ESPs) alter the total number of hemocytes in *Drosophila melanogaster*. Oregon-R adult flies were divided into male and female groups and then injected with 3 ng of ESPs from *S. carpocapsae* nematodes or PBS as a control treatment. Hemocyte counts in (**a**) males and (**b**) females were evaluated three hours post-injection (*P < 0.01; One-way ANOVA).

Hemocytes are the source of PO activity initiating the melanization pathway in *D. melanogaster* ^36^. Therefore, we examined whether *S. carpocapsae* ESPs affect the circulating hemocyte population by extracting hemolymph from adult flies and quantifying hemocyte numbers at one hour post injection. We found that male flies injected with the nematode ESPs had significantly lower number of free-floating hemocytes compared to PBS-injected males (Fig. 6a). In sharp contrast, females treated with the nematode molecules exhibited significant increase in hemocyte counts compared to their male counterparts (Fig. 6b). These results indicate that *S. carpocapsae* nematode infection factors interfere with the *D. melanogaster* cellular immune response in a sex-specific manner.

## Discussion

In order to complete their life cycle, EPNs must become activated and undergo morphological changes to commandeer and consume the resources of the infected insect host. Specifically, nematodes go from having a sealed mouth and collapsed pharynx to a fully expanded mouth and pharyngeal bulb. This transition often occurs immediately after exposure to host tissues but takes place in a nonsynchronous manner across an entire colony ^31^. The success of *S. carpocapsae* life cycle depends on this structural change in order to gain the ability to feed, therefore it would be a significant hindrance from an evolutionarily standpoint to presume that the nematode predominately relies on the release of toxins and virulence factors from its mutualistic bacteria to fulfill its most basic biological need. Yet, the release of the mutualistic bacteria into the insect hemocoel occurs a few hours after the host barriers are breached by the nematodes, which indicates that the parasites must employ their own set of pathogenic strategies ^37,38^. Furthermore, the initial containment of the bacteria within the nematode and the temporal delay in the secretion of the bacterial toxins weakens the original notion of the mutualistic bacterial partners being the primary tactical approach of the nematode-bacterial complex and instead it presumably forms a supportive strategy of the EPN’s arsenal.

The production of ESP factors from the EPN *S. carpocapsae* has previously been reported. Non-activated nematodes hardly secrete any proteins while both axenic and symbiotic nematodes equally secrete an abundance of venom proteins once activated ^29,31^. Monitoring the proteomic profiles over several hours of being activated in the hemolymph has identified time-based changes over the course of the simulated infection and determined between 6 and 30 h as the necessary optimal duration for approximately 20 IJs to produce ESPs with a lethal concentration of 20 ng per single adult fly ^31^. ESPs collected beyond this time become decreasingly less toxic with the final time point of 54 h showing no signs of toxicity despite maintaining similar protein concentrations, demonstrating that the venomous proteins produced by the nematodes exist at a relatively low abundance ^31^. This previous result may support our observation that injection of *S. carpocapsae* ESPs into adult flies causes an instantaneously lethal effect compared to a natural infection during which insect death occurs after 2–3 days of nematode infection. In other words, the injection treatment bypasses the necessary length of time for the EPNs to manufacture and secrete the lethal dose. More interestingly, it has been shown that non-activated IJs have higher gene expression of 88 out of the 472 venom proteins compared to activated IJs, suggesting that these venomous proteins may be synthesized earlier during the host seeking stage and then quickly released following cuticular invasion when the parasites reach the insect hemolymph ^34^.

*Heterorhabditis bacteriophora* is another EPN and its ESPs have also been shown to alter the host immune response when injected into adult *D. melanogaster*. Axenic nematodes activated with *Manduca sexta* hemolymph plasma cause a significant suppression in the transcriptional expression of the antimicrobial peptide encoding gene *Diptericin* ^39^. This result contrasts with our results that *S. carpocapsae* ESPs significantly induce *Diptericin* expression at the same time point. This discrepancy may indicate that different EPN species employ distinct strategies to deal with the host antimicrobial activity during the early stages of infection ^40^. The increased mortality observed in adult flies injected with lethal doses of *S. carpocapsae* ESPs may be attributed to the high expression of *Diptericin*, and presumably other antimicrobial peptide genes, induced by the exacerbated activation of the Imd pathway resulting in an uncontrolled inflammatory response and consequently the host’s demise.

Previous work has begun to isolate and characterize some of the protein compounds within the *S. carpocapsae* ESP mixture to better understand the intricacies of how these individual components target specific insect host defenses ^34^. Among the isolated molecules, different types of serine protease inhibitors (serpins) have been identified. Nematode serpins are imperative in boosting the pathogenicity of the parasites because they can inhibit the function of host serine proteases that regulate insect host homeostasis and incite innate immune cascades ^41,42^. Specifically, serpins *Sc-SRP-6* and *Sc-KU-4* have been isolated from the *S. carpocapsae* ESPs and shown to inhibit digestive enzymes, the formation of hard clots, and hemocyte aggregation and nodulation ^33,43,44^. Because the insect PPO cascade is also tightly regulated by serine proteases ^45,46^, it is a plausible target for *S. carpocapsae* secreted serpins which may suppress the PO activity and subsequent melanization response at the site of injury.

The suppression of PO activity in wild-type flies is not surprising, since the PO cascade is a rapid immune response in *D. melanogaster* and therefore it forms a prime target for inactivation by microbial invaders and nematode parasites ^47–49^. Also, the discrepancy in PO activity levels between Oregon and *w1118 D. melanogaster* lines can be due to the overall variation in immune capacity between reference laboratory fly lines, as previously demonstrated ^50^. Previous work has assessed the contributions of the three *PPO* genes in *D. melanogaster* larvae carrying single, double, and triple mutations and found that all three *PPO* genes are important in the anti-nematode response to *S. carpocapsae* infection yielding decreased survival rates of the PPO mutants compared to background controls ^35^. By injecting the *S. carpocapsae* ESPs into adult flies, we further concluded that these survival trends observed in larvae are also consistent in the adult stage. In our experiments, we find that both *PPO1* and *PPO3* mutant flies are sensitive to injection of *S. carpocapsae* ESPs, which supports our previous findings that functional *PPO* genes are not only important for the *D. melanogaster* host defense against EPNs (containing or lacking their mutualistic bacteria), but also against potent effector molecules produced by the parasites during infection.

Observing the progression of melanization in wild type adult flies, we noticed that males treated with *S. carpocapsae* ESPs experience a diminished and delayed melanization response around the injection site compared to both ESP treated females and males treated with PBS. We tested differences in melanization between males and females and not in PO activity because melanin deposition is the last step of the cascade and enzyme activity in *D. melanogaster* is affected by a complex network of serine proteases and their inhibitors ^36,51^. Sex differences in the melanization response led us to examining the impact of *S. carpocapsae* ESPs on hemocyte levels between males and females as they are associated with the activity of the PPO cascade ^52,53^. We hypothesized that if nematode ESPs were to negatively impact the *D. melanogaster* cellular immune response especially in males, then these flies would suffer substantial reduction in hemocyte levels. Indeed, we find that males treated with the *S. carpocapsae* infection factors did contain a considerably lower number of hemocytes than the control treated counterparts, which is in line with the melanization results. These findings support the notion that *S. carpocapsae* ESPs possess multifunctional properties as they are capable of interfering with various aspects of the insect host immune system and these effects occur during the early stages of nematode infection.

**Figure 7:**
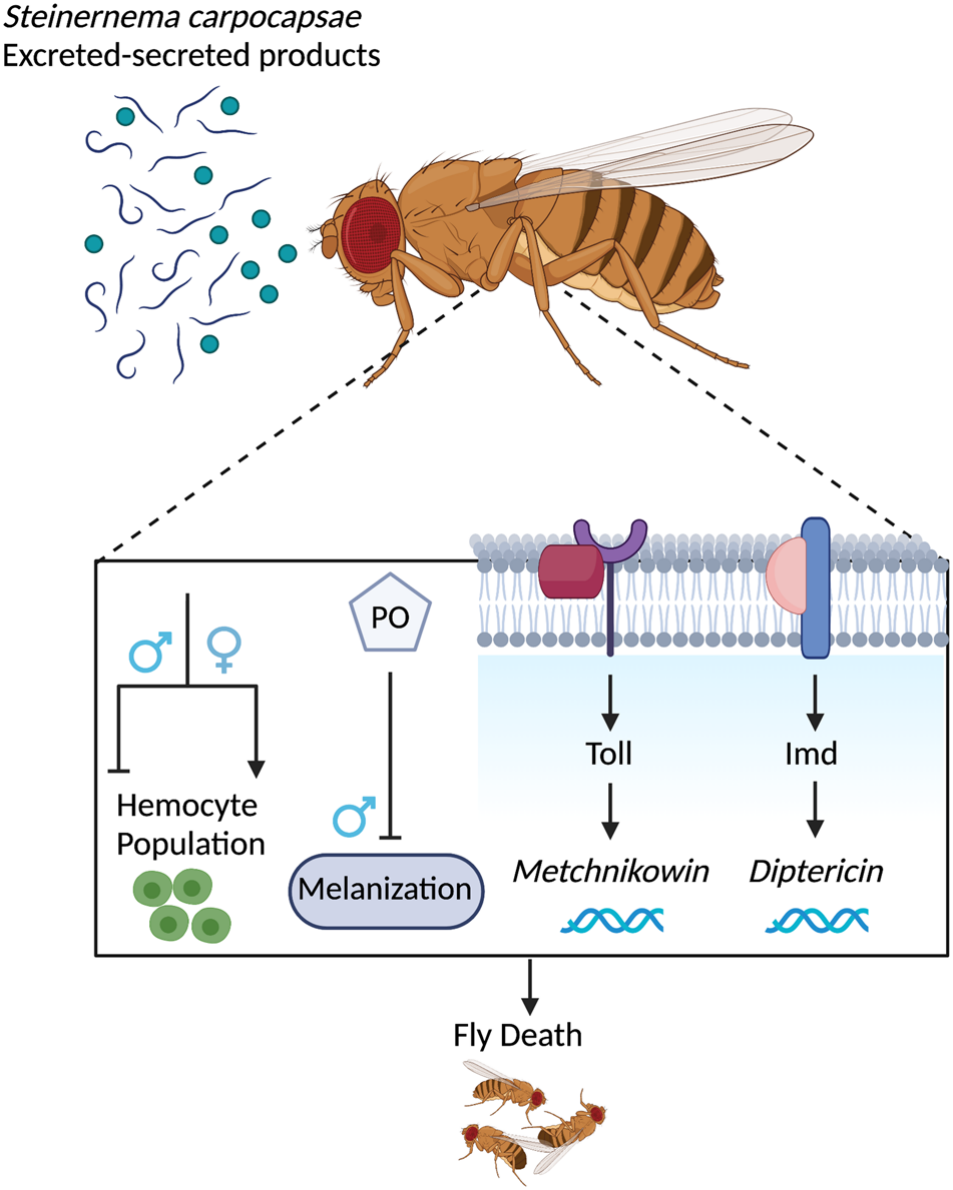
Impact of the *Steinernema carpocapsae* nematode excreted/secreted products (ESPs) on the *Drosophila melanogaster* immune response. Summary of the effects *S. carpocapsae* ESPs on the *D. melanogaster* immune system. Injection of the nematode infection factors reduces fly survival rates, activates the Immune Deficiency (Imd) and Toll signaling pathways, reduces the phenoloxidase (PO) enzyme activity, delays the melanization response in male flies, and alters hemocyte numbers in male and female individuals. All figure drawings were made using BioRender.

In conclusion, *S. carpocapsae* ESPs are pathogenic to *D. melanogaster* and able to significantly enhance Imd and Toll signaling activities in a manner that may negatively affect the fitness of adult flies through increased expression of antimicrobial peptides that could lead to a pathological inflammation response. In addition, the nematode infection factors impair the PO and melanization responses (the latter especially in males) and interfere with the cellular immune response by modifying the level of circulating hemocytes in both male and female flies (Fig. 7). Next steps could include the use of Imd and Toll mutants to identify the pathway components involved in relaying these amplified signals when flies are exposed to nematode infection factors and explore whether these processes can impact the overall host homeostasis when inhibited. Also, *S. carpocapsae* ESPs decrease the melanization response and hemocyte levels in male flies compared to females. This may be part of an evolutionary protective strategy that has preferentially selected the survival of female flies due to physiological burdens of rearing energetically expensive eggs while conversely sperm is inexpensive. This hypothesis opens up avenues for future research to identifying other sex-linked host variances in relation to interaction with pathogen effector molecules.

## Methods

### Fly stocks

All *D. melanogaster* stocks were maintained at 25 °C with a 12-h light cycle. They were fed on a diet that consisted of soy-based cornmeal (Meidi Laboratories) supplemented with yeast to outcompete the growth of unwanted microbes. All experiments were conducted with adult flies of approximately 10 days old.

### Nematode excreted/secreted products

They were isolated and prepared in a similar manner to the ESPs from the EPN *S. carpocapsae* parasitic stage, as previously described ^43^. Briefly, 22.5 million nematodes were induced for 18 h in insect tissue homogenate, washed three times with 0.8% NaCl and transferred to a fresh Tyrode’s solution. After 4 h of incubation, ESPs were filtered through a 0.2 µm cellulose acetate filter and concentrated with a 3 kDa MWCO centrifugal filter (Millipore) to 150 µl. Twenty µl of concentrated ESPs were used in SDS-PAGE analysis. The concentration of the isolated nematode ESPs was adjusted to 746 μg/ml.

### Fly survival assays

Survival experiments were conducted using *D. melanogaster* Oregon-R adult flies (approximately 10 days old). While anesthetized with carbon dioxide, fly injections were administered to the mesothorax region using a Drummond Nanoject III Programable Nanoliter Injector. The attached needle consisted of a pulled glass capillary filled with mineral oil. Each experiment was divided into three treatment groups and one control group receiving either 18.2 nl of *S. carpocapsae* ESPs at 1× (4.25 μg/μl), 1/5× (0.85 μg/μl), 1/10× (0.43 μg/μl), or 1/25× (0.17 μg/μl) dilutions or PBS as a negative control. Experimental groups included 10 adult flies (five males and five females) with two biological replicates each. Following injection, flies were transferred into plastic vials containing instant *Drosophila* medium (Carolina Biological) and housed in a separate incubator under the same conditions outlined for the laboratory fly stocks. Fly survival was assessed at 1-h intervals until the 6th-hour mark and then observed every 24 h for a week. Three independent experiments were used for statistical analysis in GraphPad Prism 5 software using the log-rank (Mantel-Cox) method with 95% CI.

### Expression of *Drosophila* signaling pathway genes

Based on the survival data, the dilution of 1/25× (3 ng) was selected as the working concentration to inject into flies with the purpose of analyzing changes in gene expression. Adult Oregon-R flies of approximately 10 days old were injected with either 18.2 nl of ESPs or PBS, and the treated individuals were collected at 0-, 6-, and 24-h time points and stored at −80 °C. Total RNA was extracted from each sample containing two male and two female flies through homogenization in TRIzol reagent (Ambion, Life Technologies) with two replicates for each time point. RNA concentration was normalized to 1,000 ng/μl, and cDNA was generated with the High-Capacity cDNA Reverse Transcription Kit (Applied Biosystems). Gene expression analysis was conducted on a CFX96 Real-Time System, C1000 Thermal Cycler (Bio-Rad). The cycle protocol was 95 °C for 2 min, 40 repetitions of 95 °C for 15 s followed by 61 °C for 30 s, and then one round of 95 °C for 15 s, 65 °C for 5 s, and finally 95 °C for 5 s. Each reaction contained a final volume of 20 μl qPCR mix comprised of 10 μl GreenLink No-ROX qPCR Mix (BioLink), 40 ng of cDNA template, forward and reverse primers at a final concentration of 200 nM and 1.2 μl sterile deionized water. Primers for the following genes were used: *Diptericin* (F: 5′-GCTGCGCAATCGCTTCTACT-3′; R: 5′-TGGTGGAGTTGGGCTTCATG-3′), *Defensin* (F: 5′-CGCATAGAAGCGAGCCACATG-3′; R: 5′-GCAGTAGCCGCCTTTGAACC-3′), *Drosomycin* (F: 5′-GACTTGTTCGCCCTCTTCG-3′; R: 5′-CTTGCACACACGACGACAG-3′), *Metchnikowin* (F: 5′-TCTTGGAGCGATTTTTCTGG-3′; R: 5′-AATAAATTGGACCCGGTCTTG-3′), *TotA* (F: 5′-GAAGATCGTGAGGCTGACAAC-3′; R: 5′-GTCCTGGGCGTTTTTGATAA-3′), *TotM* (F: 5′-GCTGGGAAAGGTAAATGCTG-3′; R: 5′-AGGCGCTGTTTTTCTGTGAC-3′), *Daw* (F: 5′-GGTGGATCAGCAGAAGGACT-3′; R: 5′-GCCACTGATCCAGTGTTTGA-3′), *Dpp* (F: 5′-CCTTGGAGCCTCTGTCGAT-3′; R: 5′-TGCACTCTGATCTGGGATTTT-3′). Primers for the housekeeping gene *RpL32* were F: 5′-GATGACCATCCGCCCAGCA-3′; R: 5′-CGGACCGACAGCTGCTTGGC-3′. Gene expression from the RT-qPCR experiments was analyzed in accordance with the 2^−ΔΔCT^ method ^54,55^. dCt values were collected from four independent trials with two technical replicates per sample and assessed with a one-way ANOVA and Bonferroni post-test using GraphPad Prism 5 software. ddCt values were graphed with standard error bars shown.

### Phenoloxidase activity

*Drosophila melanogaster* Oregon-R and *w1118* lines were subdivided into two groups and injected with either 18.2 nl of the 1/25 × dilution (3 ng) of *S. carpocapsae* ESPs or PBS (negative control). One hour post injection, 20 adult flies from each treatment group were collected (10 male and 10 females) into a Pierce Spin Column (10 μM) (ThermoFisher) and incubated on ice for 10 min. Protease inhibitor (Sigma-Aldrich) was diluted in 0.1 M sodium phosphate buffer (pH 7.4) to 2.5 × per the manufacturer’s protocol, and then 20 μl and five 4 mm glass beads were added to the spin column (Thermo Scientific) and centrifuged at 10,000 rpm for 20 min at 4 °C. The supernatants were transferred to a new microcentrifuge tube containing 10 μl of 2.5 × protease inhibitor on ice. Protein concentrations of extracted hemolymph were normalized with a Pierce BCA Protein Assay Kit (Thermo Fisher) to 15 μg in 40 μl aliquots. Using a 96-well flat bottom plate (Greiner Bio-One), each reaction well contained 160 μl of fresh L-DOPA solution (20 mM in 0.1 M sodium phosphate buffer pH 7.4), 40 μl of 5 mM CaCl_2_, and 40 μl of extracted hemolymph plasma. A blank of PBS with CaCl_2_ and L-DOPA was used as a negative control. The plate was shielded from light and incubated for 30 min at 37 °C, and then analyzed on a Synergy HTX Mutli-mode Reader (BioTek). During the run, the plate was incubated at 29 °C and absorbance was measured at 492 at a frequency of 2-min intervals with a total run time of 30 min. Absorbance values were converted to μM of dopachrome based on the molar extinction coefficient, where ε = 3700 M^−1^ cm^−1^ ^56^. The experiment was repeated three times and each experiment involved biological duplicates and technical triplicates. Two tailed t-tests were performed using the GraphPad Prism 5 software.

### Survival of prophenoloxidase fly mutants

Survival experiments were conducted with *D. melanogaster* lines *w1118* (background) and *PPO1* (Bloomington stock number 56204)*, PPO2* (Bloomington stock number 56205)*, PPO3* (Bloomington stock number 68386) mutants following the same injection protocol outlined for the survival of the wildtype flies. Each experimental group, consisting of 10 adult flies (five males and five females approximately 10 days old), received either 18.2 nl of the 1/25 × dilution (3 ng) of *S. carpocapsae* ESPs or PBS (negative control). The flies were observed for the first 6 h at 1-h intervals and then every 24 h over the course of one week. Statistical analysis of the data was performed in GraphPad Prism 5 using the log-rank (Mantel-Cox) method with 95% CI.

### Melanization response

Flies from the *D. melanogaster* Oregon-R line were separated into experimental groups containing 10 male or 10 female adults (approximately 10 days old) with two biological replicates. Flies were injected with either 18.2 nl of the 1/25 × dilution (3 ng) of *S. carpocapsae* ESPs or PBS as negative control, as described above. Three hours post treatment, the injection site was examined visually under a stereoscope to monitor the formation of melanin spots, and images were taken through the lens of a compound microscope (Olympus CX21) at 25× magnification. Melanization levels were categorized according to the degree of melanin synthesis (None, Light, Moderate, or High). The flies were continually observed at 6- and 24-h time points to document the progression of melanin intensity. Statistical analysis of the results obtained from three independent experiments was performed using two-way ANOVA, followed by a Bonferroni’s post-test using GraphPad Prism 5 software.

### Total hemocytes count

Flies from the *D. melanogaster* Oregon-R line were separated into experimental groups containing 10 male or female adults (approximately 10 days old) with biological duplicates. Flies were injected with either 18.2 nl of the 1/25 × dilution (3 ng) of *S. carpocapsae* ESPs or PBS (negative control). At the 3-h time point, fly hemolymph was extracted, and samples were loaded on a hemocytometer. Total numbers of circulating hemocytes were counted and averaged across five technical replicated using 40× magnification of a compound microscope (Olympus CX21). Statistical analysis was performed on the results from three separate experiments using one-way analysis of variance (ANOVA) in GraphPad Prism 5 software.

## Supplementary Information

Supplementary Information.

## Data Availability

The datasets generated during and/or analyzed during the current study are available from the corresponding author on reasonable request.
