# Supplementary material for: Excreted secreted products from the parasitic nematode Steinernema carpocapsae manipulate the Drosophila melanogaster immune response
Source: Sci Rep. 2022 Aug 20;12:14237. doi: 10.1038/s41598-022-18722-7 (PMC9392720; doi:10.1038/s41598-022-18722-7)
Supplement: Supplementary file 1 — Supplementary Information. [file 41598_2022_18722_MOESM1_ESM.pdf]

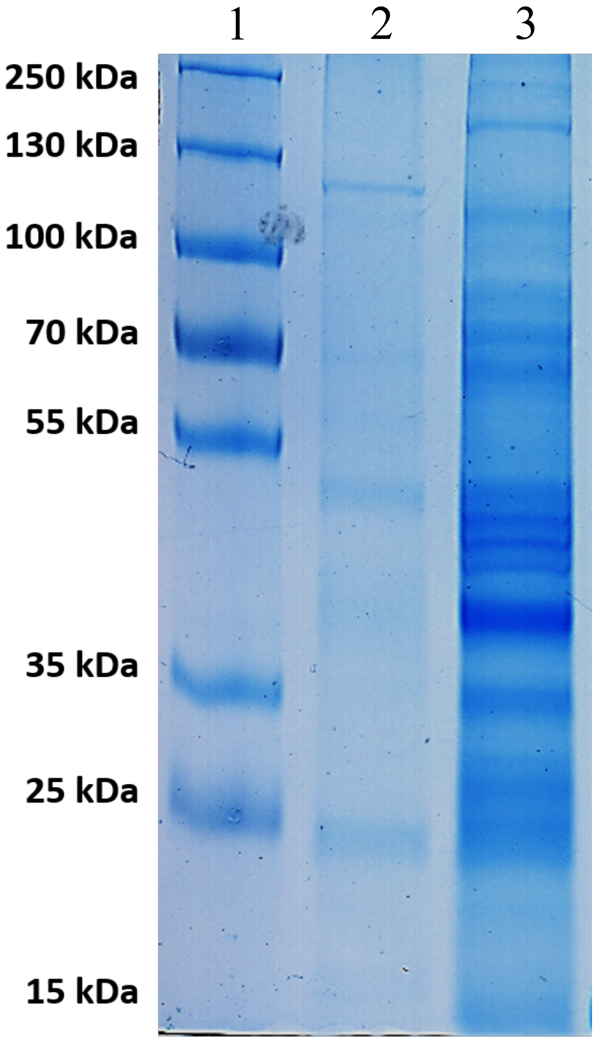

Supplementary Figure 1. SDS-PAGE analysis of the *Steinernema carpocapsae* excreted/secreted products (ESPs). Protein molecular weight marker (Plus Prestained Protein Ladder, 15 to 250 kDa, Thermo Scientific) (Lane 1), ESPs of *S. carpocapsae* induced for 18 hours in insect tissue homogenate prior to concentration (Lane 2), ESPs of *S. carpocapsae* induced for 18 hours in insect tissue homogenate after concentration (Lane 3). Picture of part of an uncropped protein SDS-PAGE gel.
